# HALP score as a prognostic marker for overall survival in advanced pancreatic cancer

**DOI:** 10.3389/fonc.2025.1542463

**Published:** 2025-04-29

**Authors:** Nazan Demir, İvo Gökmen, Yasemin Sağdıç Karateke, Ayşegül İlhan, Fatih Yıldız, Duygu Bayır Garbioğlu, Bülent Yıldız

**Affiliations:** ^1^ Department of Medical Oncology, Sultan I. Murat Public Hospital, Edirne, Türkiye; ^2^ Department of Medical Oncology, Mehmet Akif Ersoy Public Hospital, Çanakkale, Türkiye; ^3^ Department of Medical Oncology, Eskisehir Osmangazi University Faculty of Medicine, Eskisehir, Türkiye; ^4^ Department of Medical Oncology, Etlik City Hospital, Ankara, Türkiye; ^5^ Department of Medical Oncology, Dr. Abdurrahman Yurtaslan Oncology Training and Research Hospital, Ankara, Türkiye; ^6^ Department of Medical Oncology, Zonguldak Bulent Ecevit University Faculty of Medicine, Zonguldak, Türkiye

**Keywords:** HALP score, overall survival, advanced pancreatic cancer, gastrointestinal cancers, prognostic index

## Abstract

**Introduction:**

Pancreatic cancer is the leading cause of cancer-related deaths worldwide and most of the patients diagnosed at an advanced stage. Clinicians need simple, effective, and repeatable tools to predict the prognosis. This study aimed to evaluate the relationship between the HALP score and prognosis in patients with advanced pancreatic cancer.

**Methods:**

Patients diagnosed with advanced pancreatic cancer at three centers in Turkey between 2009 and 2023 were included in this retrospective study. Demographic features, blood parameters, treatment received, treatment responses, and survival were recorded.

**Results:**

227 patients were included in the study. The median overall survival (OS) for the entire cohort was 10.4 months. The median OS was 8.7 months in the low-HALP group and 11.2 months in the high- HALP group. Patients in the low-HALP group had a significantly shorter median OS than those in the high-HALP group (log rank p=0.001).

**Conclusion:**

The HALP score is a reliable and practical tool that can be utilized in clinical practice to predict prognosis in patients with advanced pancreatic cancer.

## 1 Introduction

Pancreatic cancer is one of the leading causes of cancer-related deaths worldwide. Although curative resection remains the only definitive treatment, the majority of patients are diagnosed at a locally advanced or metastatic stage, precluding surgical intervention. Even if R0 resection is achieved, the 5-year overall survival (OS) rate is approximately 30% in node-negative patients, dropping to as low as 10% in node-positive patients (1). Unfortunately, the recurrence, as well as postoperative morbidity and mortality, remain high.

Chronic inflammation plays a crucial role in the etiopathogenesis of cancer. Additionally, cancer itself is a significant cause of systemic inflammation. With recent advancements in our understanding of the tumor microenvironment, the role of chronic inflammation in tumor biology and metastasis has become more apparent. Biological changes induced by chronic inflammation can be detected by routine biochemical tests. Indexes such as the systemic inflammatory index and nutritional index have been developed based on this principle, and their utility in predicting prognosis in various cancer types has been demonstrated (2, 3). Similarly, in pancreatic cancer, the scales and ratios derived from biochemical parameters have been evaluated for their ability to predict OS and progression-free survival (PFS).

The HALP(hemoglobin, albumin, lymphocyte, and platelet) score, which is calculated using hemoglobin, albumin, lymphocyte, and platelet levels, is one such index that has been studied in several cancers, including renal cell carcinoma, lung cancer, and gastric cancer (4–6). It was initially investigated for its preoperative prognostic significance in gastric cancer patients and calculated as 'Hemoglobin (g/dl) x albumin(g/dl) x lymphocyte count (/µl) x platelet count (/µl) (7). It was also analysed together with sarcopenia to assess the prognosis of patients with operated pancreatic cancer.

Low HALP score and sarcopenia shown to be associated with increased post-operative complications and decreased OS (8).

The relationship between changes in components of HALP score and cancer was demonstrated in many studies. Anemia is observed in approximately 40-64% of cancer patients and has been associated with tumor hypoxia, apoptosis resistance, induction of tumor cell growth, and treatment resistance (9). Thrombocytosis, a common finding in many cancer patients, is linked to malignancy through mechanisms such as TGF-β-mediated suppression of natural killer cell function and activation of intracellular pathways that enhance metastasis and survival of malignant cells (10). Lymphocytes, a key component of immunity, are also affected in cancer, with lymphopenia being associated with poorer treatment responses in metastatic patients (11). Albumin, frequently used to assess nutritional status in cancer patients, is shown to correlate with performance status and OS when combined with lymphocyte levels in the prognostic nutritional index (12, 13).

In this study, we aimed to evaluate the effectiveness of the HALP score in predicting the prognosis in patients with advanced pancreatic cancer.

We believe that our research will contribute to the literature by aiding in the prediction of survival in pancreatic cancer patients, identifying high-risk patients in advance based on their prognosis, and facilitating the management of follow-up and treatment processes by clinicians.

## 2 Materials and methods

### 2.1 Data collection, assessments, and follow-up

This study was approved by the Ethics Committee of Eskisehir Osmangazi University Faculty of Medicine (protocol no: ESOGÜ-GOKAEK 2023-386) on January 16, 2024.

**Figure 1:**
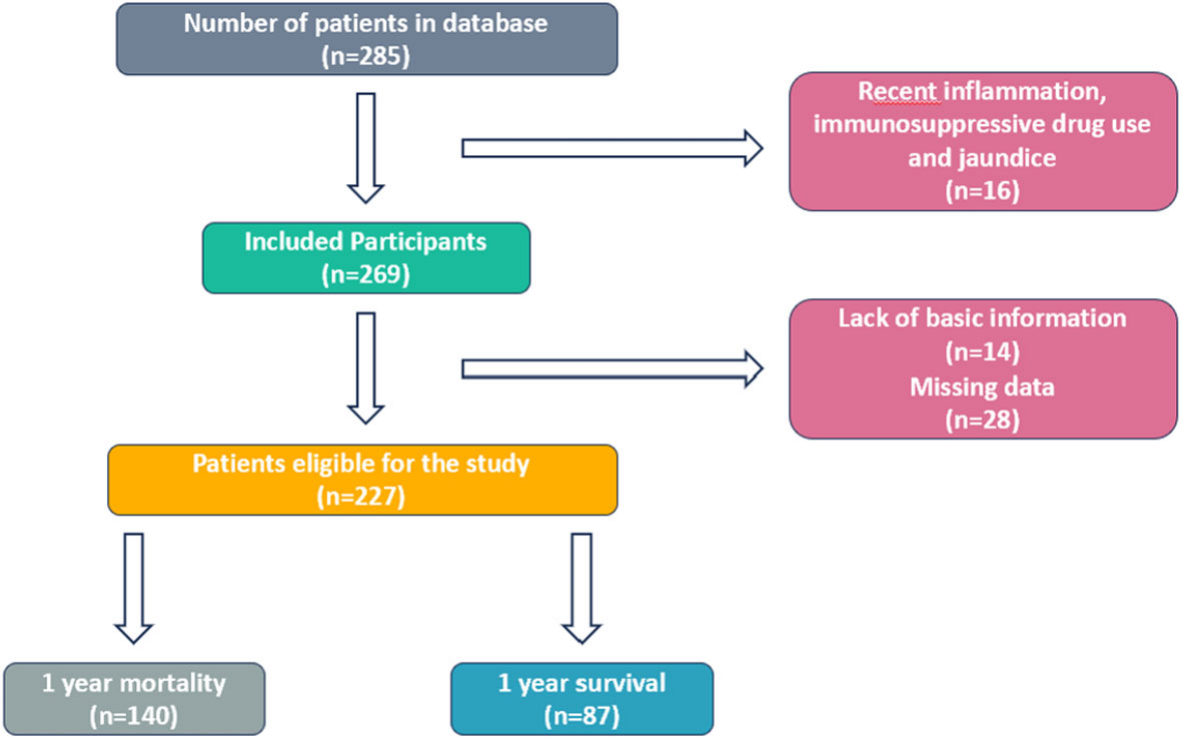
Consort diagram of patients.

A total of 227 patients who were diagnosed with pancreatic cancer via histopathological confirmation following biopsy and presented to the oncology outpatient clinics of Eskisehir Osmangazi University, Ankara Dr. Abdurrahman Yurtaslan Oncology Hospital, and Edirne Sultan I. Murat Public Hospital between March 2009 and March 2023 were included in the study. The selection process of the patients included in the study is shown in **Figure 1**.

Patients data were obtained through outpatient clinic files and hospital automation systems. Patients with endocrine pancreatic tumors, pancreatic cystic neoplasms, hematological or autoimmune diseases, active infection, biliary obstruction or those using immunesupressive drugs were excluded from the study. For *de novo* metastatic patients, data at the time of diagnosis were analyzed, whereas for patients who became metastatic during follow-up, data from the time metastasis was detected were evaluated.

Demographic data included age at diagnosis, sex, Eastern Cooperative Oncology Group Performance Status (ECOG PS), and disease stage. Clinical and treatment data included surgical status, presence of metastasis, time and location of metastasis, number of metastatic sites, chemotherapy regimens administered, start and end dates of treatments, clinical response, date of last follow-up, and date of death. Laboratory parameters evaluated included hemoglobin, red cell distribution width (RDW), neutrophil, lymphocyte, white blood cell count (WBC), platelet count, carcinoembryonic antigen (CEA), carbohydrate antigen 19-9 (CA 19-9), albumin, bilirubin levels, and lactate dehydrogenase (LDH). The HALP score was calculated from these laboratory values using the following formula:


$$\begin{array}{c} \text{HALPscore}=[\text{Hemoglobin}\left(\text{g}/\text{dl}\right)\times \text{Albumin}\left(\text{g}/\text{dl}\right)\\ \times \text{Lymphocytecount}\left(/µ\text{l}\right)]/\text{Plateletcount}\left(/µ\text{l}\right) \end{array}$$

Disease staging was performed according to the TNM staging system, incorporating tumor size (T), lymph node involvement (N), and metastasis (M), based on imaging obtained from computed tomography (CT), magnetic resonance imaging (MRI), or positron emission tomography (PET). Follow-up and treatment responses were evaluated using the Response Evaluation Criteria in Solid Tumors (RECIST 1.1) (14).

The primary endpoint of the study was OS from the time of metastatic diagnosis. OS was calculated as the time from the diagnosis of metastatic pancreatic cancer to death or the date of the last follow-up. Patients with partial or complete responses were included in the calculation of the objective response rate (ORR), while patients achieving stable disease, partial response, or complete response were included in the calculation of the disease control rate (DCR).

### 2.2 Statistical analysis

All statistical analyses were performed using IBM SPSS (Statistical Package for the Social Sciences) version 23.0 (IBM Corp., Armonk, NY, USA). Optimal cut-off values for predicting mortality were determined using receiver operating characteristic (ROC) curve analysis and the Youden index. Continuous variables were expressed as mean ± standard deviation (SD) or median (interquartile range, IQR), depending on data distribution. Patients were categorized into two groups (low and high) based on the HALP score cut-off values.

Differences between groups were analyzed using chi-square tests for categorical variables and the Mann- Whitney U test for continuous variables that were not normally distributed. Kaplan-Meier survival analysis was employed to estimate OS, with the log-rank test used to compare survival curves between the groups. Cox proportional hazards regression analysis was conducted to evaluate the relationship between OS and independent variables, such as demographic features, tumor characteristics, and treatment regimens. A p-value of less than 0.05 was considered statistically significant.

## 3 Results

### 3.1 Patient characteristics and survival outcomes

In the study population, the median follow-up period from the initial diagnosis of pancreatic cancer was 12.4 months, during which 219 patients died. Among the 227 metastatic pancreatic cancer patients, the median OS from the initiation of first-line metastatic treatment was 10.4 months (95% CI: 9.1–11.8 months), with survival rates of 38.2%, 7.4%, and 1.7% at 1, 2, and 3 years, respectively.

The mean age at diagnosis was 61 years (SD ±9.6; range: 33–83), and the ECOG performance score was most commonly 0 (27%) or 1 (60.4%). The primary tumor location was predominantly in the pancreatic head (64.3%). At the time of diagnosis, 67.8% of the patients were metastatic, and 65.6% had two or more metastatic sites. The most common metastatic sites were the liver (78%) and the lungs (30.8%). Elevated CA 19-9 levels were observed in 69.2% of patients at diagnosis (median: 1498 U/mL). The general characteristics of the 227 patients are summarized in **Table 1**.

**Table 1:** Demographic characteristics of the patients.

		*Total n,%*	*Low HALP Score Total n,%*	*High HALP Score Total n, %*	*P*
Number of patients, n (%)		227 (100)	103 (45.4)	124 (54.6)	
Median age, years (range)		61 (32-81)	62 (34-81)	59.5 (32-81)	0.629
Elderly, n (%)	< 65 years old	152 (67)	65 (63.1)	87 (70.2)	
	≥ 65 years old	75 (33)	38 (36.9)	37 (29.8)	0.321
Sex, n (%)	Female	87 (38.3)	49 (47.6)	38 (30.6)	0.007
	Male	140 (61.7)	54 (52.4)	86 (69.4)	
ECOG PS, n (%)	0	63 (27.8)	25 (24.3)	38 (30.6)	
	≥ 1	164 (72.2)	78 (75.7)	86 (59.4)	0.301
Primary tumor localization, n (%)	Head	146 (64.3)	78 (75.7)	68 (54.8)	**0.005**
	Body	63 (27.8)	20 (19.4)	43 (34.7)	
	Tail	18 (7.9)	5 (4.9)	13 (10.5)	
Metastatic condition	Non-metastatic	79 (34.8)	40 (38.8)	39 (31.5)	0.199
at initial diagnosis, n (%)	(Recurrent disease)				
	*De novo* metastatic	148 (65.2)	63 (61.2)	85 (68.5)	
Metastatic region, n (%)	Liver	177 (78)	80 (77.7)	97 (78.2)	0.920
	Lymph nodes	119 (52.4)	63 (61.2)	56 (45.2)	0.017
	Lung	70 (30.8)	32 (31.1)	38 (60.6)	0.945
	Peritoneum	64 (28.2)	40 (38.8)	24 (19.4)	**0.002**
	Bone	25 (11)	13 (12.6)	12 (9.7)	0.527
Number of metastatic regions, n (%)	<2	78 (34.4)	27 (26.2)	51 (41.1)	**0.024**
		149,65.9			
	≥ 2		76 (73.8)	73 (58.9)	
Chemotherapeutic agent, n (%)	Single agent	51 (22.5)	30 (29.1)	21 (16.9)	**0.012**
	Doublet regimen	71 (31.3)	36 (35.0)	35 (28.2)	0.011
					0.003
	Triplet regiment	105 (46.3)	37 (35.9)	68 (54.8)	
Level of carbohydrate antigen 19-9	Normal	70 (30.8)	30 (29.1)	40 (32.3)	0.666
	Abnormal	157 (69.2)	73 (70.9)	84 (67.7)	

Tumors located in the pancreatic head (p = 0.005), peritoneal metastases (p = 0.002), and ≥2 metastatic regions (p = 0.024) were more frequent in patients with low HALP scores, suggesting a potential link between low HALP and higher tumor burden or aggressive disease. Triplet chemotherapy was more commonly administered in patients with high HALP scores (p = 0.003), possibly reflecting better performance status in this group.

In our study patients with low HALP scores more frequently had tumors located in the pancreatic head, extensive lymph node involvement, and peritoneal metastases. Single-agent chemotherapy was also more prevalent in this group, suggesting that the HALP score may be associated with tumor location, metastatic burden, and treatment tolerance.

The chemotherapy regimens and response rates used in this study are summarized in **Table 2**. Among 227 patients, PR was observed in 55 patients (23.3%), SD in 76 patients (33.5%), and CR in only 2 patients (0.9%), and the remaining 94 patients (41.4%) had progressive disease (PD). ORR in the entire population was 24.2%, and DCR was 57.7%. The highest ORR was achieved with mFOLFIRINOX (36.3%) and nab-paclitaxel + gemcitabine (35.7%), whereas gemcitabine monotherapy had an ORR of 7.5%.

**Table 2:** First-line treatment features.

Median duration of treatment, months, (range), n (%)		Total n, (%)
Chemotherapy regimen	mFOLFIRINOX	102 (44.9)
	Gemcitabine+nab-paclitaxel	14 (6.2)
	Gemcitabine+Cisplatin	37 (16.3)
	Gemcitabine	53 (23.3)
	FOLFOX	17 (9.3)
Best Response Rate	Partial Response	55 (24.2)
	Stable Disease	76 (33.5)
	Progressive Disease	96 (42.3)

### 3.2 Determination of optimal HALP score cut-off value

**Figure 2:**
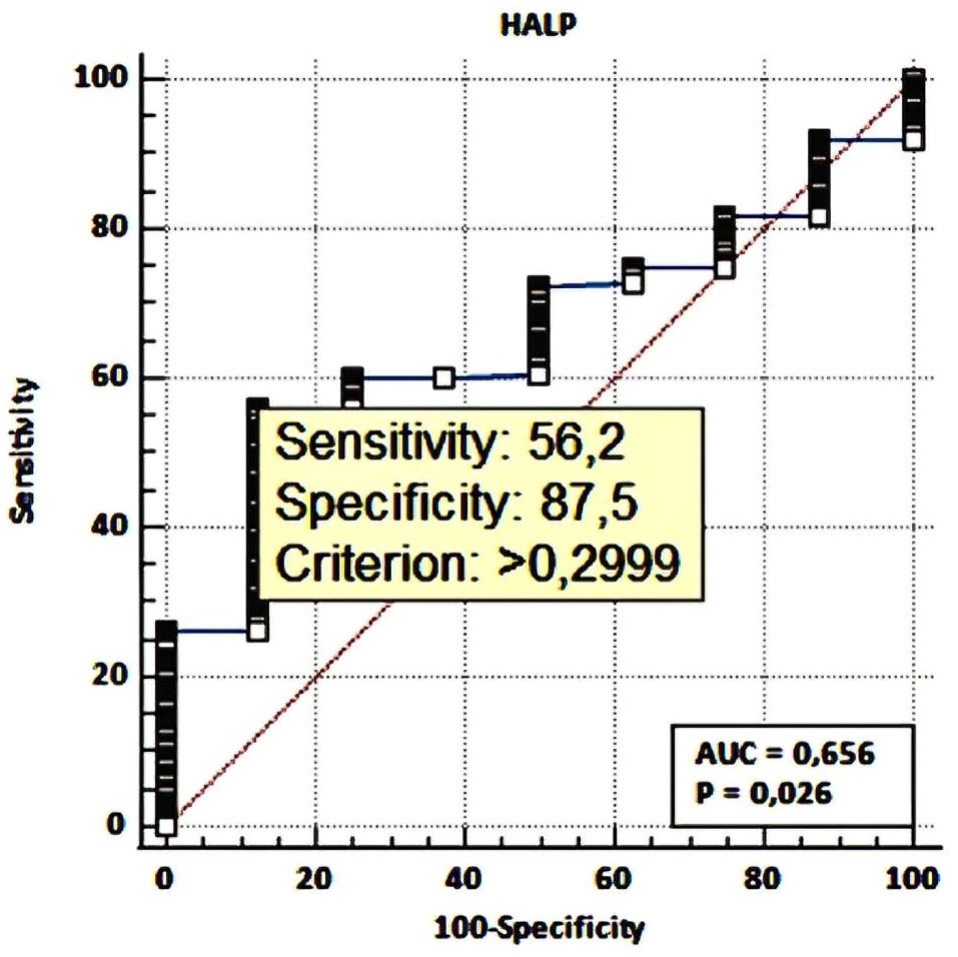
The HALP score cut-off value for survival prediction, sensitivity and specifity.

The optimal cut-off value for the HALP score for predicting survival was determined using the ROC curve and the Youden index. The HALP score cut-off value for survival prediction was identified as 29.90 (AUC: 0.656; sensitivity: 56.2%; specificity: 87.5%; p=0.026) (**Figure 2**). When compared to the mean cut-off value (31.32; range: 8.3–89.34) determined through Cox regression analysis, the Youden index-based cut-off showed better prognostic value than the mean-based cut-off (HR: 0.693; 95% CI: 0.529–0.906; p=0.002; HR: 0.713; 95% CI: 0.545–0.934; p=0.013). Based on this cut-off value, patients were divided into two groups: High-HALP (>29.90, n=124, 54.6%) and Low-HALP (≤29.90, n=103, 45.4%).

### 3.3 Analysis of prognostic risk factors and survival

In univariate analysis, clinical factors significantly associated with OS included an ECOG performance score of ≥1 (HR: 1.556; 95% CI: 1.151–2.105; p=0.004), primary tumor location in the pancreatic head (HR: 1.334; 95% CI: 1.005–1.771; p=0.046), *de novo* metastatic disease at diagnosis (HR:1.38; 95% CI: 1.040-1.84; p=0.026), a HALP score equal to or below the mean (HR:0.69; 95% CI: 0.529-0.906; p=0.002), and the use of single-agent chemotherapy in first-line metastatic treatment (p<0.001) (**Table 3**).

**Table 3:** Univariate and multivariate analyses results including factors that may affect overall survival.

			Univariate		Overall Survival		
		*HR*	*CI (%)*	*P*	*Median*	*CI (%)*	*P*
Elderly, n (%)	< 65 years old				10.9	9.6-12.2	0.580
	≥ 65 years old	1.312	0.989-1.741	0.060	9	7.5-10.4	
Sex, n (%)	Female				11.7	9.3-14.2	0.094
	Male	1.266	0.961-1.668	0.093	9.2	7.7-10.7	
ECOG PS, n (%)	0				11.9	9.4-14.5	**0.004**
	≥ 1	1.556	1.151-2.105	0.004	9.1	7.9-10.3	
Primary tumor localization, n (%)	Head	Ref.		0.103	9.4	8-10.8	0.100
	Body	0.716	0.526-0.973	**0.033**	12.9	10.5-14	
	Tail	0.916	0.544-1.540	0.739	8.2	6-10.4	
Metastatic condition at initial diagnosis, n (%)	Non-metastatic at diagnosis (recurrent disease)				11.1	9.1-13.1	**0.025**
	Metastatic (*de novo*)	1.386	1.040-1.847	**0.026**	9.5	8.1-10.9	
Number of metastatic regions, n (%)	<2				10.8	9.3-12.3	0.643
	≥ 2	1.066	0.806-.1.411	0.653	9.1	7.6-10.5	
Chemotherapeut ic agent, n (%)	Single agent	Ref.		**0.000**	6.8	5.9-7.7	0.000
	Doublet regimen	1.715	1.209-2.432	**0.003**	10.8	9.3-12.3	
	Triplet regiment	0.813	0.597-1.109	0.192	11.7	9.7-13.7	
Level of carbohydrate antigen 19-9	Normal				10.2	7.9-12.4	0.652
	Abnormal	1.068	0.801-1.425	0.653	9.8	8.4-11.2	

Tumors located in the pancreatic head (p = 0.005), peritoneal metastases (p = 0.002), and ≥2 metastatic regions (p = 0.024) were more frequent in patients with low HALP scores, suggesting a potential link between low HALP and higher tumor burden or aggressive disease. Triplet chemotherapy was more commonly administered in patients with high HALP scores (p = 0.003), possibly reflecting better performance status in this group.

In multivariate analysis, high HALP score was independently associated with improved overall survival (HR: 0.685, 95% CI: 0.524–0.891; p = 0.034). Similarly, receiving combination chemotherapy reduced the risk of mortality compared to single-agent therapy (HR: 0.534, 95% CI: 0.382–0.746; p < 0.001), and absence of lung metastases was also associated with better survival (HR: 0.648, 95% CI: 0.472–0.889; p = 0.007).

## 4 Discussion

Numerous studies have been conducted to predict prognosis in metastatic pancreatic cancer, and several prognostic factors have been identified (15–17). However, there remains a need for biomarkers that are easily applicable in daily clinical practice. Hemoglobin levels are known to affect the survival of patients with malignancies, and increased mortality observed in anemic patients (18). Albumin, a negative acute-phase protein synthesized in the liver, and reduced levels of it is associated with malnutrition, inflammation, and metabolic changes caused by cancer cells. Hypoalbuminemia is a significant prognostic marker associated with poor survival in cancer patients (19). The immune system also plays a crucial role in prognosis. Lymphocytes help inhibit tumor progression through cytolysis and other mechanisms. Lymphopenia in peripheral blood leads to impaired immune function and poor prognosis in malignancies (20). Platelets support angiogenesis, tumor growth, and cellular motility via growth factors, and thrombocytosis has been associated with poor OS in cancer patients (21). Therefore platelet count is included as a divisor, rather than a multiplier, in the HALP score formula (22).

In this context, HALP score stands out as a significant prognostic index that evaluates both nutritional status and immune system function (23–25). Limited studies on pancreatic cancer have demonstrated an association between the HALP score and OS. This study aimed to contribute to the literature by evaluating the effectiveness of HALP score in predicting the prognosis in patients with advanced pancreatic cancer.

**Figure 3:**
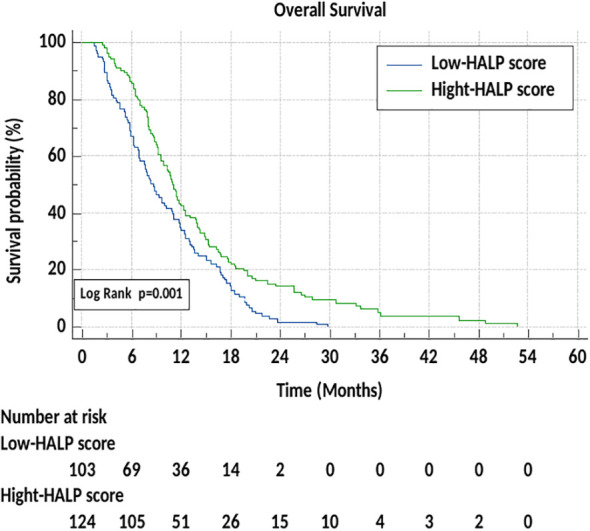
Overall survival according to Low and High-HALP score.

In our study, a low HALP score was associated with reduced OS compared to a high HALP score in patients with advanced pancreatic cancer. In the multivariate Cox regression analysis, the HALP score remained an independent prognostic factor for OS. Patients with a high HALP score had a 31.5% reduced risk of death compared to those with a low HALP score (HR: 0.685, 95% CI: 0.524–0.891; p = 0.034). This finding strengthens the prognostic utility of the HALP score, indicating that it retains significance even after adjusting for other clinical and pathological factors such as performance status, metastatic burden, and treatment regimen.The median OS was 8.7 months in the low HALP group (95% CI: 7.1–10.5) and 11.2 months in the high HALP group, with a statistically significant difference (log rank p=0.001) (**Figure 3**). A high HALP score was found to be a good prognostic indicator in advanced pancreatic cancer, consistent with findings in the literature (5, 23, 26). Across all patients, the median OS was 10.4 months (95% CI: 9–11.8), with survival times and patient characteristics similar to those reported in previous studies (6, 26).

The prognostic importance of the HALP score in gastrointestinal (GI) cancers is particularly noteworthy (26–28). However, studies specifically focusing on pancreatic cancer are limited. In a study on resectable pancreatic adenocarcinoma, a high preoperative HALP score was significantly associated with longer OS and recurrence-free survival (RFS) (29). The same study reported that a low HALP score was linked to adverse prognostic factors such as lymph node metastasis, poor tumor differentiation, and higher TNM stage (29). These findings support the role of HALP score as a prognostic marker in resectable pancreatic cancer. Another study on pancreatic cancer found better OS outcomes in patients with recurrent metastatic disease compared to those with *de novo* metastatic disease (26). In our study, the median OS was 9.5 months (95% CI: 8.1–10.9) for *de novo* metastatic disease and 11.1 months (95% CI: 9.1–13.1) for recurrent metastatic disease, with a statistically significant difference (p<0.05).

Other studies on GI cancers further highlight the broader prognostic significance of the HALP score. In a study on resectable esophageal squamous cell carcinoma, data from 756 patients were analyzed, revealing that high HALP scores were significantly associated with better OS, while low HALP scores correlated with deeper tumor invasion and larger tumor sizes (30). Similarly, in a study by Chen et al. on gastric cancer patients, high HALP scores were significantly associated with smaller tumor sizes, earlier stages, and longer OS, whereas low HALP scores were linked to lymph node metastasis and poor differentiation (7).

Also in colorectal cancers, low HALP score reported to be significantly associated with shorter OS and cancer-specific survival (CSS). Furthermore, the HALP score was noted to assist in distinguishing between malignant and benign causes of bowel obstruction (31, 32). A study on biliary tract cancer involving 418 patients demonstrated that high HALP scores were associated with longer OS, while low HALP scores were linked to higher TNM stages and unfavorable clinical features (33).

The prognostic significance of the HALP score also documented in other cancer types. Gao et al., in a retrospective study in stage III and IV non-small cell lung cancer (NSCLC) patients, found that lower HALP scores were associated with poorer OS and progression-free survival (PFS) (24). Similarly, in tongue cancer and pharyngeal cancer, low HALP scores were associated with shorter OS (25, 34). These findings are consistent with the results of our study.

Studies in the literature show that the cut-off values for the HALP score vary depending on the cancer type and the statistical methods used for analysis. For instance, in esophageal cancer, the cut-off values range between 31.8 and 38.8, whereas studies on gastric cancer often report higher cut-off values, such as 56.8. For colorectal cancers, the cut-off values fall between 15.5 and 26.5, and for gastrointestinal stromal tumors, the cut-off value has been reported as 43.62. In studies on resectable pancreatic cancer, a higher preoperative HALP score cut-off value of 44.8 has been identified (35).

In our study, the HALP score cut-off value was determined to be 29.9. This finding highlights the need for cancer type- and patient-specific considerations when interpreting the HALP score. Additionally, differences in cut-off values may originate from the use of various statistical methods, such as X-tile and ROC analysis. X-tile typically identifies higher cut-off values, while ROC analysis offers lower cut-off values, balancing sensitivity and specificity. Overall, the literature suggests an average HALP score cut-off value of approximately 31.2 (35). Therefore, the HALP score should be adapted to the cancer’s type and its stage, making it a tailored prognostic tool for different patient groups.

In our study, the HALP score emerged as a significant parameter reflecting not only prognosis but also the biological behavior of the tumor and treatment tolerance in pancreatic cancer patients. Patients with low HALP score were significantly more likely to have tumors located in the pancreatic head and a higher frequency of lymph node metastasis (p<0.05). Additionally, higher rate of peritoneal metastases in the low HALP score group (p<0.01) underscores the association of the HALP score with metastatic patterns.

From a treatment tolerance perspective, patients with low HALP score were more frequently treated with single-agent chemotherapy (p<0.05), indicating their ineligibility for intensive treatment regimens. Conversely, patients with high HALP score were more likely to receive intensive treatment protocols and demonstrated higher response rates. This may be attributed to the fact that patients with higher HALP scores tend to have a more stable clinical condition and blood parameters, which might lead clinicians to prioritize intensive treatment for these individuals. Vice versa the limitation in treatment intensity may be attributed to a low HALP score, which is associated with reduced performance status and OS. These findings suggest that the HALP score is not only a valuable tool for predicting survival but also a crucial parameter for guiding clinicians for decision-making processes.

The retrospective design of our study has led to certain limitations. The inclusion of both *de novo* metastatic and recurrent metastatic patients may have contributed to a heterogeneous study population, potentially limiting the generalisability of the results. Although patients presenting with jaundice or cholangitis at the time of blood sampling were excluded, it was not possible to distinguish those who had initially presented with jaundice but had normalized following intervention. Treatment-related toxicity rates, the need for dose reductions, and treatment interruptions were not evaluated due to the lack of systematic data collection across centers. To better understand the prognostic value of the HALP score, prospective studies focusing on specific stages and involving homogeneous patient groups are required. Such studies may allow the HALP score to give more consistent results with specific cut-offs in specific groups.

## 5 Conclusion

The HALP score can serve as an inexpensive, simple, and cost-effective tool for prognostic assessment in patients with advanced pancreatic cancer. In this study, we aimed to contribute to the literature by evaluating the HALP score, which has been assessed as a prognostic indicator in various diseases, in the context of advanced pancreatic cancer. Our results showed that a high HALP score was associated with longer OS in these patients. However, larger and more comprehensive studies are needed to establish a general cut-off value for pancreatic cancer.

## Data Availability

The raw data supporting the conclusions of this article will be made available by the authors, without undue reservation.
